# Sensory Evaluation of Rabbit Meat from Individuals Fed Functional and More Sustainable Diets Enriched with Freshwater *Cladophora glomerata* Macroalgal Biomass

**DOI:** 10.3390/ani13132179

**Published:** 2023-07-03

**Authors:** Monika Nutautaitė, Asta Racevičiūtė-Stupelienė, Alius Pockevičius, Vilma Vilienė

**Affiliations:** 1 Institute of Animal Rearing Technologies, Veterinary Academy, Lithuanian University of Health Sciences, Tilžės Str. 18, LT-47181 Kaunas, Lithuania; asta.raceviciutestupeliene@lsmuni.lt (A.R.-S.); vilma.viliene@lsmuni.lt (V.V.); 2 Department of Veterinary Pathobiology, Veterinary Academy, Lithuanian University of Health Sciences, Tilžės Str. 18, LT-47181 Kaunas, Lithuania; alius.pockevicius@lsmuni.lt

**Keywords:** macroalgae, sustainability, muscle fiber length, histomorphometry, physical properties, emotional response

## Abstract

**Simple Summary:**

Researchers conducted a study to investigate the use of freshwater *Cladophora glomerata* macroalgal biomass as an alternative feed material for rabbits, with a focus on improving meat quality and sustainability while meeting consumer preferences. The rabbits were fed diets with varying amounts of biomass, and the resulting meat was evaluated for physical and sensory characteristics. The findings revealed that incorporating macroalgal biomass positively impacted meat production, making it more sustainable and appealing to consumers. However, the inclusion of biomass increased moisture content and cooking losses in hind leg muscles while reducing the darkness and redness of both fresh and cooked meat, enhancing its visual appeal. Moreover, using an 8% biomass inclusion led to longer muscle fibers. Notably, evaluators reported increased happiness after tasting hind leg muscles from the same diet, as evidenced by their emotional responses. Sensory evaluations further confirmed that the taste and overall quality of the rabbit meat were deemed acceptable.

**Abstract:**

Maintaining meat quality is essential to sustainable livestock management. Therefore, identifying alternative feed materials while considering consumer acceptance is crucial. So, the aim of this study was to evaluate the effect of *C. glomerata*-biomass-supplemented feeds on rabbit muscles’ physical properties, sensory profiles, and evaluators’ emotional responses to them. A total of thirty 52-day-old weaned Californian breed rabbits were randomly allocated to one of three dietary treatments: standard compound diet (SCD), SCD supplemented with 4% *C. glomerata* (CG4), or SCD supplemented with 8% *C. glomerata* (CG8). After the 122-day-old rabbits were slaughtered, post-mortem dissection of the rabbit *Longissimus dorsi* (LD) and hind leg (HL) muscles was conducted. The physical and histomorphometric features, sensory analyses, and emotional responses to the rabbit’s muscles were determined. Study results revealed CG4 and CG8 treatments significantly increased rabbit muscle moisture, while CG8 increased cooking losses in HL muscles (*p* < 0.05). Moreover, both CG treatments reduced the darkness and redness of fresh and cooked rabbit muscles compared to SCD (*p* < 0.05). CG8 treatment compared to SCD resulted in longer LD muscle fibers (*p* < 0.05). Evaluators discovered that the average scores for each sensory description of rabbit meat are acceptable and that consuming CG8-HL muscles can increase happiness based on emotional responses. Consequently, replacing traditional feed materials in rabbit feed with *C. glomerata* can lead to not only more sustainable production but also more consumer-acceptable rabbit meat.

## 1. Introduction

With a growing population that directly correlates with increasing demand for meat [1], field-to-table processes need to be ensured, and more sustainable solutions need to be sought. An increasing number of scientific studies are being published where traditional feed materials are partially replaced by alternative ones in animals’ diets [2,3,4,5]. Scientists are increasingly turning to natural and renewable sources and trying to enhance or discover innovative strategies that would help develop more sustainable livestock management. For example, the freshwater *Cladophora glomerata* (*C. glomerata*) macroalgal biomass thrives in water bodies such as rivers. Collecting this kind of biomass has two huge benefits: firstly, it cleans up water bodies and thus increases their diversity; secondly, collected biomass can play a multifunctional role in many branches of biotechnology.

Although ensuring environmental and production sustainability is essential for preserving the global food supply, it should not be achieved at the cost of compromising quality [2]. Furthermore, the concept of meat quality is undergoing a paradigm shift, and contemporary consumers are increasingly concerned about the comprehensive aspects of meat, including its nutritional value, sensory attributes, cooking convenience, and cost-effectiveness. The sensory characteristics of meat, including its taste, odor, texture, and visual appearance, in conjunction with its nutrient composition, exert substantial influence on consumer preferences and purchasing decisions. Nerveless, it is critical to unravel the intricate interplay of multiple factors that govern meat quality and elucidate their underlying mechanisms in order to effectively enhance overall quality. Pethick et al. [6] highlighted several fundamental consumer-focused criteria for meat products’ future value propositions. According to one of them, it is recommended that products possess health-enhancing properties by incorporating high-quality protein and nutrients, including fatty acids, minerals, and vitamins that align with the requirements of a healthy diet.

The development of innovative techniques is of utmost importance to attract consumers and encourage them to perceive rabbit meat as an enticing and viable alternative to the more commonly consumed pork, beef, and poultry products. By introducing novel approaches, it becomes possible to enhance the market appeal of rabbit meat and broaden its acceptance among consumers. Additionally, it is imperative to incorporate this approach into the development of novel technologies to expand market offerings and address specific consumer demands, including convenience [7]. Addressing consumer concerns about sustainability, the utilization of alternative raw materials such as the biomass of freshwater macroalgae, such as *C. glomerata*, in rabbit feed production offers a solution that is derived from renewable sources and reduces the ecological footprint of conventional feed production. In our previous studies, we presented the potential of *C. glomerata* biomass collected from Lithuanian rivers as a more sustainable and even functional feed material by analyzing its chemical composition, fatty and amino acid profiles, as well as antioxidant activity [8,9,10]. Furthermore, after conducting a feeding trial with rabbits and replacing traditional feed materials with different dosages of *C. glomerata* biomass, we discovered that this kind of alternative feed formulation can improve the functionality of rabbit meat even further [11].

While it is important to improve the general characteristics of meat quality, it is crucial not to neglect the sensory aspects, which newly introduced feed raw materials can directly affect. Despite the growing prevalence of health-conscious consumer trends, sensory attributes encompassing flavor, odor, and visual appeal continue to play a pivotal role as key determinants of product acceptance. These sensory characteristics remain crucial aspects influencing consumer perceptions and preferences, thereby underscoring their utmost importance in evaluating overall product desirability. In general, understanding what consumers demand in terms of meat, how they perceive it, and how they prefer it is therefore critical for optimizing meat quality and production, as well as maximizing profitability for meat producers and distributors [12]. Furthermore, the value of meat products is a complex amalgamation of customer expectations that directly influence both the willingness to pay and the ultimate decision to acquire these essential food sources in the human diet [13]. As a result, the objective of this study was to assess the effect of feed supplemented with various dosages of *C. glomerata* on rabbit muscle physical properties, sensory characteristics, and the emotional responses of evaluators towards them.

## 2. Materials and Methods

### 2.1. Animals and Samples Collection

**Figure 1:**
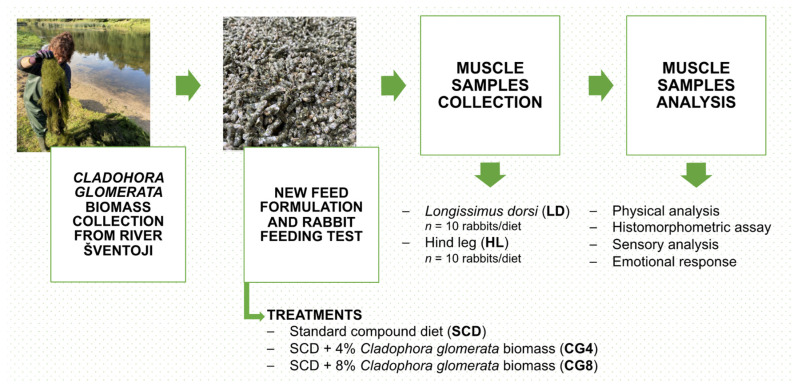
Protocol structure of the rabbit feeding trial.

The study was carried out at a local rabbit breeding facility, where the animals were housed indoors in individual cages measuring 34 cm × 34 cm × 61 cm, accommodating one rabbit per cage. The rabbits had unrestricted access to individual nipple drinkers, providing clean drinking water as well as feed bowls, ensuring optimal health conditions and performance. The building was equipped with a heating system that maintained a temperature of 19 ± 2 °C. The housing conditions adhered to the standards outlined in Council Directive 98/58/EC of 20 July 1998, which focuses on the welfare of animals kept for farming purposes. The biomass of *C. glomerata* used for feed formulation was manually collected from the Šventoji River in Lithuania, cleaned, dried, and subsequently utilized in feed production. The chemical composition of the biomass has been previously examined and reported [8,9,10]. Protocol structure from the Šventoji River to rabbit muscle analysis is presented in Figure 1.

The feeding trial was conducted using thirty male Californian breed rabbits that had been weaned at 52 days of age. The rabbits, selected based on having similar weight, were randomly allocated to three dietary treatments (*n* = 10 rabbits/diet). They were provided with a standard compound diet (SCD), SCD supplemented with 4% biomass of freshwater *C. glomerata* (CG4), and SCD supplemented with 8% biomass of *C. glomerata* (CG8), with feeding occurring twice a day *ad libitum*. The formulation of the standard compound diet was developed to fulfill the nutritional needs of growing rabbits by incorporating essential vitamins and minerals. The utilized feed ingredients and their corresponding chemical compositions can be found in the previously published research by Nutautaite et al. [11] (Section 2.1, Table S1). The nutrient composition of the diet was formulated based on the recommendations provided by the National Research Council [14].

Upon completion of the feeding trial, when the rabbits reached 122 days of age, a total of 30 rabbits (*n* = 10 rabbits/diet) were subjected to weighing, followed by an overnight period of fasting, and subsequently euthanized in accordance with standard practices. The slaughtering procedure was conducted at a rabbit farm slaughterhouse, following established protocols that align with the legal requirements set forth in the Republic of Lithuania (Order No. B1-866 of 31 October 2012, issued by the Director of the State Food and Veterinary Service) outlining the approved regulations for the care, keeping, and utilization of animals for scientific and educational purposes.

The dissection procedures of warm and chilled carcasses followed the World Rabbit Science Association recommendations [15]. The rabbit carcasses were chilled at a temperature of 4 °C for 24 h in a well-ventilated room. Subsequently, the *Longissimus dorsi* (LD) and hind leg (HL) muscles were dissected from the reference carcasses (*n* = 10 LD muscles/diet; *n* = 10 HL muscles/diet).

### 2.2. Physical Analysis of Rabbit Muscles

The moisture content in samples was determined by drying 2 g minced muscle samples in an oven (105 °C) to a constant weight. The difference between before and after drying the sample was calculated and expressed in percentages (%).

The drip loss was determined according to the methods described by Honikel [16]. In total, 25 g of the studied muscles were placed in mesh bags to reduce evaporation and hung on hooks (4 °C). The samples were reweighed after 24 h to determine the weight change. Drip loss was expressed in percentages (%).

The pressing method was applied to determine the water-holding capacity [17]. Muscle samples were weighed (300 mg) on an analytical balance and placed on ash-free filter paper. On top, a plastic plate was placed and pressed down with a 1 kg weight. It was kept for 10 min, after which the weight was removed, and the boundaries of the compressed meat were defined on the filter paper with a graphite pencil. Using the DIGIPLAN Digital Planimeter 300 planimeter (Gebrüder Haff GmbH, Pfronten, Germany), the spot area was determined, and the difference between the inner and outer spot moisture areas was calculated. Water holding capacity was expressed as a percentage (%).

The cooking loss of the LD and HL muscles was determined by employing Honikel’s method [18]. Meat samples were weighed (25 g), placed in polyethylene bags, and then cooked in a circulating water bath for 30 min at a temperature of 90 °C. The cooked samples were cooled to room temperature before being taken from the bags, drained, and weighed; the cooking loss was calculated and expressed in percentages (%) based on the change in mass before and after cooking.

At 24 and 48 h after slaughter, the pH of rabbit LD and HL muscles was determined using the Inolab 730 device (WTW GmbH, Weilheim, Germany).

Using the Chroma Meter CR-410 (Konica Minolta, Inc., Osaka, Japan), rabbits’ raw muscles at 24 and 48 h post-mortem and cooked muscles (25 min in an 80 °C water bath) were examined. The same contrast color space was used to define the color coordinates. The light reflectance mode was used to estimate the coordinates L*, a*, and b* (brightness, redness, and yellowness coordinates, respectively, on the CIELAB scale). The measurements were performed using reference light source C, whose radiation is quite similar to that of typical daylight. The equipment was calibrated using a light trap and a white standard before each measurement.

### 2.3. Histomorphometric Assay of Rabbit LD Muscles

Histomorphometric properties of 30 LD muscles (*n* = 10 LD muscles/diet) were determined. Each 1 × 1 cm piece of muscle from the same location of the LD muscle’s middle section was removed, and it was fixed in 10% neutral buffered formalin. Tissue sections of 4 mm thickness were cut using a rotary microtome Leica RM 2235 (Leica Microsystems, Nussloch, Germany) and stained with hematoxylin and eosin, followed by the standard paraffin embedding technique. Using an Olympus BX63 microscope (Olympus Corp., Tokyo, Japan), an Olympus DP72 digital camera (Olympus Corp., Tokyo, Japan), and a computer running the Image-Pro Plus application system for Windows, version 7.0 (Media Cybernetics, Inc., Bethesda, MD, USA, 2009), prepared *Longissimus dorsi* muscle histologic preparations were analyzed. LD muscle fibers’ cross-sectional areas (fiber length; 150 fibers were measured in each sample in three fields of view), as determined morphometrically, are expressed in micrometers squared (µm^2^).

### 2.4. Sensory Analysis of Rabbit Muscles

A sensory profile test was used to evaluate the sensory properties. A group of ten trained evaluators aged between 34 and 42 years participated in the test and evaluated LD and HL muscles (*n* = 10 LD muscles/diet; *n* = 10 HL muscles/diet). Among the evaluators, 80% were female and 20% were male. Evaluators were selected and trained to work according to LST ISO 8586-1 (ISO). The evaluation was closed and performed according to the requirements of LST ISO 8589 (ISO). The evaluators assessed the samples in separate sensory booths. At the initial stage of research, sensory properties were selected, based on which the samples were analyzed and compared with each other.

#### 2.4.1. The Preparation and Submission of Samples for Sensory Evaluation

Muscle samples of LD and HL were enclosed in a cooking bag and subsequently put into an HS-B20 water bath that has automatic temperature regulation (IKA Labortechnik, Staufen, Germany). The samples were cooked for 25 min at 80 °C according to the Martinez-Alvaro and Hernandez [19] method with some modifications. After that, the samples were removed from the bag, cooled to room temperature, and cut into 1.5 × 1.5 cm sample pieces. The samples prepared in this way were placed in plastic containers, covered with lids, coded, and immediately presented to the panel of evaluators. Tasteless, odorless water at room temperature was used to restore the taste receptors of the evaluators.

#### 2.4.2. The Procedure for Submitting Samples to Evaluators and Evaluation

A fully balanced randomized sampling design was used for sensory profiling (*n* = 10 LD muscles/diet; *n* = 10 HL muscles/diet). Ten samples were presented in each session, and a panel of evaluators evaluated them for 10 min before and after the break. The intensity of each feature of the test samples was assessed using a 7-point numerical scale: 1 means the feature is not felt, 4 means it is moderately expressed, and 7 means it is very strongly expressed. The sensory profile in the results is presented as an average of all evaluators’ responses.

### 2.5. Emotional Response Evaluation of Rabbit Muscles

Cooked (cooking conditions are described in Section 2.4.1) rabbit LD and HL samples were evaluated by the same 10 evaluators from a sensory assessment using FaceReader 8 software (Noldus Information Technology, Wageningen, The Netherlands) connected to a web camera (Microsoft Corporation, Redmond, WA, USA) to evaluate evaluators’ expressions of emotion (by viewing, smelling, and tasting the samples). Participants evaluated LD and HL muscles (*n* = 10 LD muscles/diet; *n* = 10 HL muscles/diet). The program is capable of recognizing and capturing eight different models or emotions of facial expressions in real time, including neutral, happy, sad, angry, surprised, scared, disgusted, and contempt, as well as calculating the valence to describe the positive or negative emotional state of the individual. Before starting the assessment, the participants were familiarised with the assessment procedure. Meat samples from different rabbit muscles were cut into pieces, coded, and submitted to the evaluators. The evaluation sequence was view, smell, and taste. The software captures the evaluator’s emotions. Between each sample, the evaluator rinsed the mouth with room-temperature water. The intensity of the evaluated emotions is measured on a numerical scale ranging from 0, indicating no expression of emotion, to 1, indicating the maximum value of the fitted model, while for valence, a scale of −1 to 1 was used. Emotional valence can be defined as a quantitative measure that assesses the emotion’s polarity from positive to negative. The emotional response in the results is presented as an average of all evaluators’ responses.

### 2.6. Statistical Analysis

SPSS for Windows, version 25.0 (IBM Corp., Armonk, NY, USA), was used to analyze the data. Before analyzing each data set, the Kolmogorov–Smirnov test was employed to determine normality. To identify any differences between treatments, a one-way analysis of variance (ANOVA) test was performed post hoc (Fisher’s least significant difference test). A *p*-value of less than 0.05 (*p* < 0.05) was considered statistically significant.

## 3. Results

### 3.1. Physical Features of Rabbits Muscles

The physical properties of different rabbit muscles are presented in Table 1. The inclusion of *C. glomerata* in the rabbit diet increased the moisture content of both muscles; the higher the *C. glomerata* dosage, the higher the moisture content observed (*p* < 0.05). The moisture content of the muscles was distributed as follows in both analyzed muscles: SCD < CG4 < CG8. Both 4% and 8% doses of *C. glomerata* had no significant impact on rabbit muscle drip loss or water-holding capacity (*p* > 0.05). However, CG8 treatment significantly increased cooking losses in HL muscles. Compared to the CG4 treatment, it was higher in HL muscles by 9.67% in CG8 (*p* < 0.05); LD muscles remained unaffected (*p* > 0.05).

**Figure 2:**
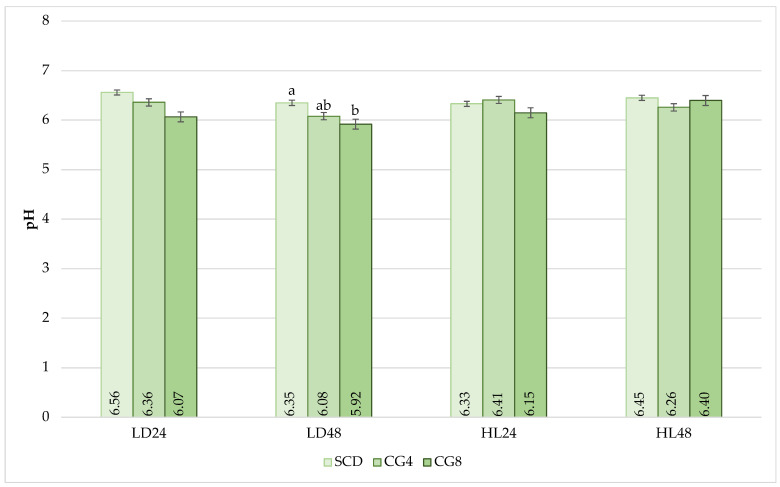
Effect of *C. glomerata* biomass supplementation in feed on pH values of rabbit muscles at 24 h (LD24 and HL24) and 48 h (LD48 and HL48) post-mortem. Note: SCD, standard compound diet; CG4, standard compound diet + 4% *C. glomerata* biomass; CG8, standard compound diet + 8% *C. glomerata* biomass. Columns of the same indicator but with different superscript letters (a,b) differ significantly (*p* < 0.05); those with ab superscript letters did not have significant differences between groups (*p* > 0.05).

The pH of individual muscles was measured at 24 and 48 h after slaughter (Figure 2). The pH remained relatively constant throughout the study periods, and the values remained without significant fluctuations. Significant differences were found only after 48 h when the pH values of SCD and CG8 LD muscles differed significantly: the pH value for 8% *C. glomerata* inclusion was 0.43 units lower compared to SCD (*p* < 0.05). In other cases, no significant impact of biomass on pH values was observed (*p* > 0.05).

**Figure 3:**
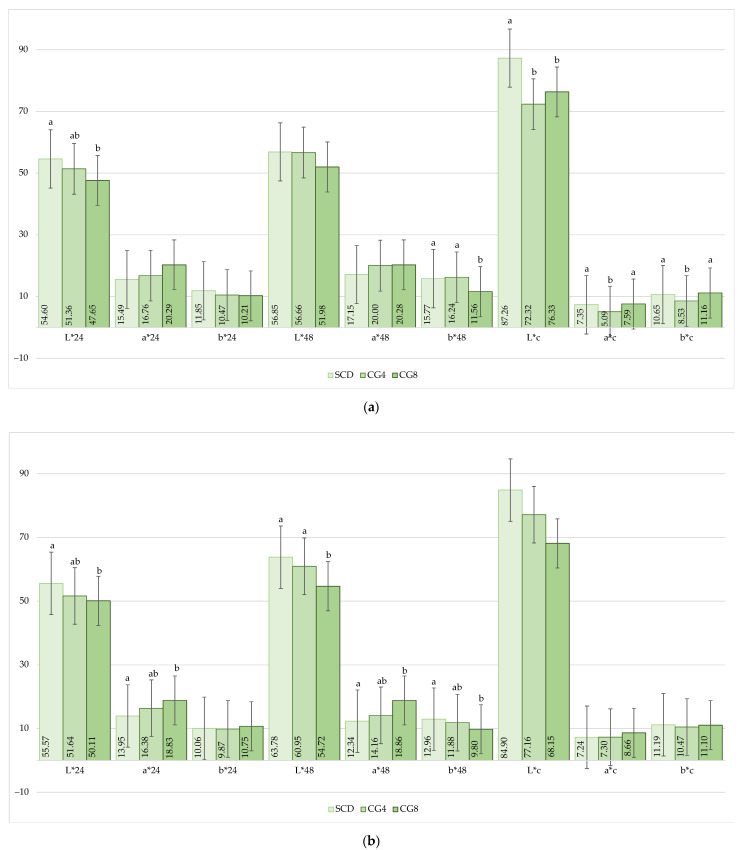
Effect of *C. glomerata* biomass supplementation in feed on the color coordinates at 24 h (L*24; a*24; b*24) and 48 h (L*48; a*48; b*48) post-mortem and after cooking (L*c; a*c; b*c): (**a**) LD muscles; (**b**) HL muscles; Note: SCD, standard compound diet; CG4, standard compound diet + 4% *C. glomerata* biomass; CG8, standard compound diet + 8% *C. glomerata* biomass. Columns of the same indicator but with different superscript letters (a,b) differ significantly (*p* < 0.05); those with ab superscript letters did not have significant differences between groups (*p* > 0.05).

The color coordinates of LD and HL muscles were measured at 24 h (L*24, a*24, b*24) and 48 h (L*48, a*48, b*48) after slaughter, as well as after cooking the rabbit meat (L*c, a*c, b*c). The results are shown in Figure 3a,b. At the starting point (24 h), the CG8 treatment had a significant impact on the L*24 coordinate of LD muscles, which was found to be 6.95 units lower compared to the SCD treatment (*p* < 0.05; Figure 3a). Similarly, at the same measuring point, the L*24 and a*24 coordinates of the HL muscles were significantly affected: L*24 decreased, and a*24 increased under the CG8 treatment compared to SCD (*p* < 0.05; Figure 3b). After 48 h, the b*48 coordinate was the lowest in the LD muscles treated with CG8; it was significantly lower by 4.21 and 4.68 units compared to SCD and CG4, respectively (*p* < 0.05). After analyzing the color coordinates of the HL muscles at 48 h, significant differences were observed among all the groups. Firstly, the L*48 coordinate was lower by 9.06 and 6.23 units under CG8 compared to SCD, while the a*48 coordinate was higher by 6.52 units compared to SCD. The b*48 coordinate was slightly lower by 3.16 units in CG8 compared to SCD (*p* < 0.05).

After cooking the rabbit meat and measuring the color coordinates, *C. glomerata* macroalgal biomass inclusion significantly affected all the measured coordinates only in LD muscles (*p* < 0.05), while on the contrary, no impact was found on the color of cooked HL muscles (*p* > 0.05). The brightest cooked meat, according to L*c, was obtained in LD muscles under SCD treatment. The lowest a*c and b*c values were determined in LD muscles treated with 4% biomass inclusion. A*c was lower in CG4 by 2.26 and 2.50 units, and b*c was lower by 2.12 and 2.63 units compared to SCD and CG8, respectively (*p* < 0.05).

### 3.2. Histomorphometric Measurements of Rabbit LD Muscles

The fiber size of the rabbit *Longissimus dorsi* (LD) muscle was measured (Table 2). Macroalgal biomass inclusion had a positive impact on muscle fiber length by increasing it; under CG8 treatment, LD fiber length increased by 7.57 μm^2^ compared to SCD and by 6.83 μm^2^ compared to CG4 (*p* < 0.05).

### 3.3. Sensory Evaluation of Rabbit Muscles

**Figure 4:**
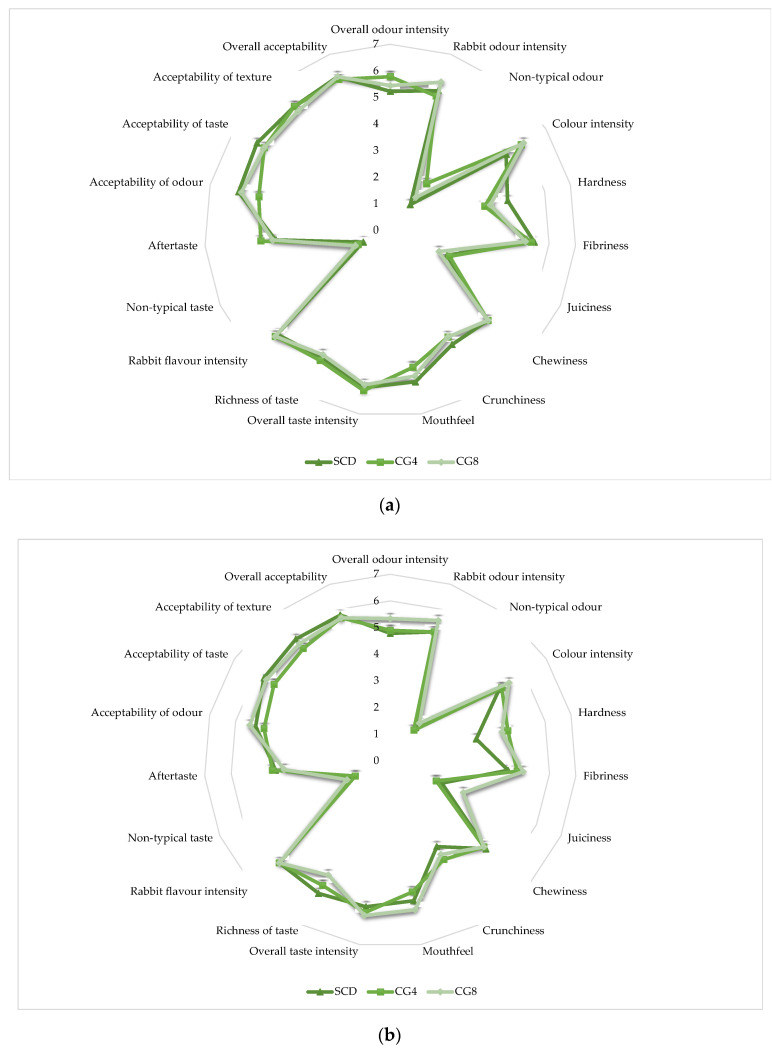
Effect of *C. glomerata* biomass supplementation in feed on sensory profile of cooked rabbit: (**a**) LD muscles; (**b**) HL muscles.

The LD and HL muscles, which were cooked prior to analysis, were independently graded using a seven-point system based on 19 criteria (Figure 4a,b). Freshwater *C. glomerata* inclusion significantly affected only two criteria in LD muscles: non-typical odor and color intensity (Figure 4a). The LD-SCD had the lowest non-typical odor, whereas according to the evaluators, the CG4 and CG8 had 1 and 0.34 points higher non-typical odors compared to the SCD, respectively (*p* < 0.05). Therefore, more intense color was expressed in CG4 (5.89 points) and CG8 (6.00 points) LD muscles, whereas slightly less intensity was observed in SCD (5.22 points) (*p* < 0.05). After the sensory assessment of HL muscles, three criteria were significantly affected: hardness, mouthfeel, and richness of taste (Figure 4b). The HL muscles under the SCD treatment exhibited significantly lower hardness compared to the CG4 and CG8 treatments, with the SCD muscles being 1.23 and 1 point less hard, respectively (*p* < 0.05). The most expressed mouthfeel after tasting rabbit meat was evaluated in CG8, and when compared to CG4, the mouthfeel was expressed more by 0.67 points (*p* < 0.05). However, 8% *C. glomerata* inclusion decreased HL muscle richness of taste in comparison to SCD, which had 0.78 points more richness of taste (*p* < 0.05).

### 3.4. Emotional Response to Rabbit Muscles

The emotional response to the rabbit muscles was evaluated visually (response to the view), by smelling it (response to the odor), and by tasting it (response to the taste) (Table 3). When analysing evaluators’ responses to the view of rabbit muscles, it mostly evoked neutral emotion. Compared to SCD, responses to the LD and HL muscles of CG4 and CG8 that evoked neutral emotion were, respectively, higher by 0.359 and 0.354 in the LD muscles and by 0.087 in the HL muscles of CG8 (*p* < 0.05). However, rabbit LD and HL muscles treated with CG4 and CG8 evoked less happy emotion compared to evaluators in SCD after viewing them (*p* < 0.05). Evaluators were sadder when viewing CG4 LD muscles, but when responding to HL muscles, *C. glomerata*-treated muscles evoked less sad emotion in CG4 and CG8 compared to SCD (*p* < 0.05). CG8-HL muscles left evaluators less disgusted than after viewing SCD (*p* < 0.05). Contempt for evaluators was higher when evaluating CG4-LD muscles and, on the contrary, when responding to HL muscles, was higher in SCD (*p* < 0.05). A negative valence after viewing rabbit muscles was observed after evaluating CG8-LD muscles and SCD-HL muscles (*p* < 0.05). None of the evaluated views of the muscles evoked anger, surprise, or fear (*p* > 0.05).

The evaluator’s reaction to the odor of rabbit meat mostly evoked a neutral emotion (Table 3). Evaluators remained more neutral after smelling CG4 and CG8 LD muscles compared to SCD (*p* < 0.05). Therefore, less neutral emotion was evoked after smelling the HL-muscles of CG4 compared to SCD and CG8 (*p* < 0.05). However, the happiest evaluators were those who evaluated the odor of SCD-treated LD and HL muscles. A sadder emotional response to odor was observed for CG8-HL muscles; it was almost two times higher compared to SCD (*p* < 0.05). Therefore, SCD LD and HL muscle odor evoked more angry emotions compared to *C. glomerata*-biomass-treated groups (*p* < 0.05). Similarly, SCD LD muscle odor elicited higher levels of surprise and fear, and HL muscle odor elicited higher levels of disgust and contempt (*p* < 0.05). A negative valence for the odor of rabbit muscles was observed after evaluating CG8-LD muscles and SCD-HL muscles (*p* < 0.05).

Evaluators also assessed one of the most important sensory properties of food: taste (Table 3). However, no significant response to the muscles of rabbits fed *C. glomerata* biomass was elicited in terms of the following emotions: neutral, sad, angry, surprised, scared, contempt, or valence (*p* > 0.05). However, evaluators expressed about seven times higher happiness levels after tasting 8% *C. glomerata*-treated rabbit HL muscles than when tasting SCD-HL muscles (*p* < 0.05). In comparison to both CG diets, CG8 left evaluators more disgusted than CG4 after tasting LD muscles (*p* < 0.05), whereas SCD-HL muscles had a nearly identical response to CG8 (*p* > 0.05).

## 4. Discussion

### 4.1. Physical Properties of Rabbit Muscles

Rabbits demonstrate exceptional attributes for meat production, characterized by a brief gestation period, abundant productivity, and remarkable feed conversion efficiency. Normally, the quality of rabbit meat is consistent [20]. The following and most important meat quality traits are distinguished: the meat’s sensory features, chemical and physicochemical composition, health-enhancing abilities, nutritional values, and safety. Moreover, the meat’s moisture content is a significant quality parameter as well [21]; it can have a direct impact on a final food product’s physical appearance (shape, color), texture, flavor, and weight, as well as aspects affecting shelf life, freshness, quality, and resistance to bacterial contamination. In our case, *C. glomerata* inclusion in rabbit feed significantly increased the moisture content of LD and HL muscles. The higher macroalgal biomass dosage in feed was determined by the higher moisture content in muscles (SCD < CG4 < CG8; *p* < 0.05). The moisture content of rabbit muscles under *C. glomerata* treatment ranged from 75.40% to 77.58% in LD muscles and from 76.45% to 77.53% in HL. Abu Hafsa et al. [22] utilized the freshwater macroalgae *Spirodela pollyrrhiza* and *Cladophora aegagropila*, which were collected from irrigation canals in Egypt, as dietary supplements for male New Zealand white rabbits. The reported results showed that the moisture content of rabbit meat in general ranged from 70.35% to 72.86%, which was slightly lower compared to our results. Therefore, it is important to note that the properties and nutritional value of macroalgae can be directly affected by seasonality, so the results compared to those of other scientists do not necessarily have to coincide. Dietary moisture content is also linked to water-holding capacity and drip loss [23]. Water holding capacity, for example, is an important quality indicator for determining meat’s economic worth since it measures the meat’s ability to retain the tissue water present in its structure [24]. Most water loss from meat is caused by structural factors such as shrinkage of myofibrils, breakdown of cell membrane structure, integrity of the cytoskeleton, progression of spaces between cells, and the development of a net between sarcoplasmic and myofibrillar proteins [25,26,27]. Nevertheless, *C. glomerata* inclusion had no significant impact on rabbits’ LD and HL muscles’ drip loss or water-holding capacity. Only one affected feature of muscles remained—cooking loss—which was significantly higher in HL muscles when dietary treatment contained 8% macroalgal biomass, compared to a two-times lower dosage (CG4). Water loss holds significant importance in the food industry as it influences the technological yield of the cooking process. The majority of water loss during cooking results from the expulsion of juices due to protein denaturation and muscle contraction, and it can be directly influenced by the cooking temperature [28]. Moreover, cooking causes an increase in the stiffness of the myofibrillar structure of meat due to protein denaturation, which is related to higher water loss [25], which can explain the results obtained in this study.

Meat pH has a substantial impact on meat quality since it affects water-holding capacity, color, tenderness, and even shelf life [29,30]. Rabbits’ muscle pH fell uniformly after muscle maturation at 24 and 48 h post-mortem. Only one significant difference was detected between SCD and CG8 when comparing LD muscle pH values at 48 h; a significantly lower pH was obtained in CG8 compared to SCD. One of the major determinants of the water-holding capacity is the rate at which pH falls, which is usually connected with post-mortem anaerobic muscle glycolysis. However, *C. glomerata* biomass in rabbit feed had no effect on the water-holding capacity of muscles; hence, no concrete mechanism between pH and water-holding capacity could be found in our study. Generally, the drop in pH in normal muscle post-mortem ranges from 7.0 to 5.5 (in our case, pH ranged from 6.56 to 5.92 in LD and from 6.41 to 6.26 in HL) due to shrinkage in the myofilament lattice and water ejection, with the resultant loss of water from the meat via dripping, exudate, or purging [25].

When buying meat, consumers first pay attention to its color, which is an optical characteristic that is directly impacted by muscle tissue structure and histological pattern. Regardless of heme pigment content, meat color may be affected by the depth of light absorption and its reflection, as seen by the diversity of color brightness measurements [31]. Therefore, acidity can play a crucial role and directly impact meat color as well [32,33]. As well as the pH of the rabbits’ different muscles, the color coordinates were determined 24 and 48 h after slaughter. When compared to standard compound diet-treated rabbit muscles 24 h after slaughter, LD and HL muscles treated with CG8 were significantly brighter in the L* coordinate, and HL was also significantly redder in the a* coordinate. So according to our results, the higher the pH, the darker the muscles observed. Analyzing muscles after 48 h revealed that 8% *C. glomerata* inclusion slightly reduced redness in LD muscles compared to the remaining treatments. After 48 h, HL muscles became even darker under CG8 treatment; the muscles were more yellow but less red compared to SCD and CG4. The darker color was also reflected in pH, which increased by several units in SCD and CG8 after 48 h of maturation, but the difference was not significant. This mechanism of action, where pH can directly determine L* (brightness) parameters, was obtained not only during our study but also established and confirmed by other scientists [32,34]. Another factor that directly determines the color of the meat and its stability is the antioxidant activity or the increased number of antioxidants [35,36]. It is important to note that higher macroalgal biomass inclusion reduced the redness of the muscles at almost all points post-mortem. This could be based on our prior findings that freshwater *C. glomerata* macroalgal biomass collected from Lithuanian rivers plays a significant role in the identified antioxidant effect [9].

Cooked meat’s appearance can be impacted not just by pH, as with fresh meat, but also by the source of the meat, packaging, storage conditions, fat accumulation, flavorings, and preservation methods [37]. Depending on the type of muscle, adequate cooking leads to color changes to off-white, grey, or brown tones. Myoglobin is the primary pigment responsible for color changes in meat, whereas the final color is determined by the degree of ferrihemochrome production, which is determined by the initial proportionality of the myoglobin as well as the final concentration of undenatured oxymyoglobin or deoxymyoglobin [38]. *C. glomerata* inclusion in rabbit feed had an impact only on cooked LD muscle color coordinates; HL muscle color coordinates were not affected. A biomass dosage of 4% in feed significantly brightened rabbits’ cooked LD muscles; these kinds of muscles were significantly less yellow and red compared to SCD and CG8.

### 4.2. Fibre Length of Rabbit LD Muscles

Muscle fiber length is a valuable economic feature since it directly influences meat yield and quality [39]. In recent research, the macroalgal biomass of *C. glomerata* increased *Longissimus dorsi* muscle fiber length to 59.09 μm^2^, whereas in SCD it was only 51.52 μm^2^. In general, the number of muscle fibers remains steady during embryonic development, although muscle mass increases after birth due to skeletal muscle fiber hypertrophy [40]. This suggests that increasing muscle fiber width or length has a significant impact on muscle yield. According to Sarsenbek et al. [41], the length and density of muscle fibers can have a direct impact on the tenderness of meat tissue, which is also an important factor in evaluating muscle tenderness. While the exact mechanism behind the observed effect of *C. glomerata* biomass on muscle fiber length is not completely understood, it is possible that the nutritional properties of the biomass are involved. The macroalgal biomass may contain specific nutrients or bioactive compounds that play a role in the development and maintenance of muscle fibers. For instance, the freshwater *C. glomerata* biomass used in the study was found to be rich in proteins and minerals, which could potentially support muscle growth and development [10]. So, according to histomorphometric measurements of rabbit LD muscles, we can state that 8% *C. glomerata* inclusion stimulates muscle growth and development by increasing the fiber length of the *Longissimus dorsi*.

### 4.3. Sensory Profile of Rabbit Muscles

Meat quality has typically been evaluated by sensory factors such as appearance, texture, odor, and flavor, and humans cook meat to increase both its digestibility and sensory properties. Tenderness, on the other hand, remains one of the most highly valued features of cooked meat [42,43,44,45]. Rabbit meat is described as exceptionally tender by consumers. However, evaluators found HL muscles under *C. glomerata* treatments to be significantly harder. The LD and HL muscles of rabbits were sensory evaluated according to 19 criteria. Algal biomass treatment had an effect not only on HL muscle hardness but also on the non-typical odor and color intensity in LD muscles and in HL muscles, and *C. glomerata* altered mouthfeel and richness of taste. The greatest impact on non-typical odor in LD muscles was caused by feed supplemented with 4% *C. glomerata*, and the intensity of the color rose was uniformly affected with increasing biomass dose in the diet. Rabbits’ HL muscles had a much more expressed mouthfeel after tasting muscles under the CG8 diet but were evaluated as having a less rich taste compared to those under the remaining diets. The remaining sensory aspects were not affected by the addition of *C. glomerata* at 4% or 8% in rabbit feed. In their review of macroalgae in rabbit nutrition, Al-Soufi et al. [42] stated that available scientific research suggests that a macroalgae-supplemented rabbit diet could further improve the intrinsic properties of meat and enhance its stability. Although there were some substantial discrepancies between the standard compound diet and the *C. glomerata* treatments in our study, the findings indicate that the average scores for each sensory descriptor are adequate for the sensory profile of rabbit meat.

### 4.4. Emotional Response to Rabbit Muscles

It has been proposed that there is a close relationship between consumption conditions and how consumers feel [46]. Expressed feelings are regarded as fundamental modulators of food perception, food liking, and overall satisfaction with human eating experiences [47]. Consumer evaluation has been broadly applied to assess food and its products’ acceptability and quality, especially meat. Torrico et al. [48] highlighted two key objectives of consumer food testing: (1) understanding the consumer’s acceptability and liking preferences, and (2) understanding the consumer’s intent to buy and pay for the product. A FaceReader software gadget was used to measure facial expression while viewing, smelling, and tasting different rabbit muscles. This type of innovative technology enables the analysis of facial expressions over time by recording the physical responses of the eyes, mouth, brows, and head. When analyzing evaluators’ responses to the view, odor, and taste of rabbit muscles that were fed experimental *C. glomerata* diets, they mostly evoked a neutral emotion, which was the predominant expression among all treatments. Therefore, evaluators were significantly less happy and even sadder after viewing LD muscles when rabbits were fed 4% biomass in their feed. On the other hand, HL muscles evoked less sadness and less disgust after viewing samples from rabbits treated with 8% *C. glomerata*. The contempt of evaluators was higher when evaluating CG4-LD muscles, and, on the contrary, when responding to HL muscles, it was higher in the standard compound diet. None of the evaluated views of the muscles significantly evoked anger, surprise, or fear. The evaluator’s reaction to the odor of rabbit meat mostly evoked a neutral emotion, similar to that after viewing them. Therefore, standard compound diet-treated LD and HL muscles evoked more happy and angry emotions at the same time compared to *C. glomerata*-biomass-treated groups. Similarly, SCD-LD muscle odor elicited higher levels of surprise and fear, and SCD-HL muscle odor elicited higher levels of disgust and contempt. Evaluators also assessed one of the most important sensory properties of food: taste. However, no significant response to the muscles of rabbits fed *C. glomerata* biomass was elicited in terms of the following emotions: neutral, sad, angry, surprised, scared, contempt, and valence. Therefore, evaluators expressed about seven times higher happiness levels after tasting 8% *C. glomerata*-treated rabbit HL muscles. In comparison to both *C. glomerata*-supplemented diets, 8% inclusion left evaluators more disgusted than 4% after tasting LD muscles. Organoleptic characteristics heavily influence consumer purchasing decisions and meat acceptance. As a result, unconscious consumer responses via biometrics combined with self-reported responses provide a deeper understanding of the acceptability of meat products. Despite the reactions to the odor and viewing samples, the evaluators were several times happier when tasting the HL muscles of rabbits that were fed with an 8% biomass dose in feed, which is a significantly positive result of this study. However, it is important to note that the rabbit’s muscles were only cooked without any additional seasoning, which may have influenced the acceptability of the samples among evaluators.

## 5. Conclusions

Altering traditional feed materials with alternative freshwater *C. glomerata* macroalgal biomass in rabbit feed production can result in not only more sustainable production but also more consumer-acceptable rabbit meat. However, inclusions of 4% and 8% biomass can significantly increase rabbit muscle moisture, and 8% in the feed can significantly increase cooking losses in HL muscles. Moreover, *C. glomerata* can reduce the darkness and redness of fresh (24 and 48 h post-mortem) and cooked rabbit muscles, which can be more appealing to consumers. According to the research findings, including 8% of *C. glomerata* biomass in rabbit feed can lead to a significant increase in the length of muscle fibers in LD muscle. Despite several substantial discrepancies between SCD and CG treatments, evaluators claim that the average scores for each sensory descriptor are appropriate and acceptable for the sensory profile of rabbit meat. Notwithstanding their reactions to the odor and viewing the samples, CG8-HL rabbit muscles can increase happiness, according to the evaluators’ emotional responses after tasting them.

## Figures and Tables

**Table 1:** Effect of *C. glomerata* biomass supplementation in feed on the physical characteristics of rabbit muscles.

		Diet ^2,3,4^				
Item (%)	Muscle ^1^	SCD	CG4	CG8	SEM ^5^	*p-*Value ^6^
Moisture	LD	74.72 ^a^	75.40 ^b^	77.58 ^c^	0.26	0.000
	HL	74.37 ^a^	76.45 ^b^	77.53 ^c^	0.31	0.000
Drip loss	LD	1.99	2.46	2.65	0.29	n.s.
	HL	2.18	2.97	1.84	0.51	n.s.
Water holding capacity	LD	62.93	64.91	70.99	3.32	n.s.
	HL	70.91	69.88	64.98	4.01	n.s.
Cooking loss	LD	21.37	24.75	22.37	2.81	n.s.
	HL	22.99 ^ab^	21.37 ^a^	31.04 ^b^	3.74	0.041

Note: ^1^ LD, *Longissimus dorsi*; HL, hind leg. ^2^ SCD, standard compound diet; CG4, standard compound diet + 4% *C. glomerata* biomass; CG8, standard compound diet + 8% *C. glomerata* biomass. ^3^ The means with distinct superscript letters (a–c) in a row differ significantly (*p* < 0.05). ^4^ Means with ab superscript letters in a row did not have significant differences between groups (*p* > 0.05). ^5^ SEM, standard error of the means. ^6^ n.s., not significant (*p* > 0.05).

**Table 2:** Effect of *C. glomerata* biomass supplementation in feed on LD muscle fiber length.

		Diet ^2,3^				
Item	Muscle ^1^	SCD	CG4	CG8	SEM ^4^	*p-*Value
Fibre length (μm^2^)	LD	51.52 ^a^	52.26 ^a^	59.09 ^b^	2.38	0.002

Note: ^1^ LD, *Longissimus dorsi*. ^2^ SCD, standard compound diet; CG4, standard compound diet + 4% *C. glomerata* biomass; CG8, standard compound diet + 8% *C. glomerata* biomass. ^3^ The means with distinct superscript letters (a,b) in a row differ significantly (*p* < 0.05) ^4^ SEM, standard error of the means.

**Table 3:** Effect of *C. glomerata* biomass supplementation in feed on the emotional response to cooked rabbit muscles.

		Diet ^2,3,4,5^				
Evoked Emotion	Muscle ^1^	SCD	CG4	CG8	SEM ^6^	*p-*Value ^7^
		Response to the view Scale 0–1				
Neutral	LD	0.458 ^a^	0.817 ^b^	0.812 ^b^	0.065	0.000
	HL	0.783 ^a^	0.826 ^ab^	0.870 ^b^	0.035	0.017
Happy	LD	0.434 ^a^	0.044 ^b^	0.164 ^b^	0.083	0.000
	HL	0.285 ^a^	0.075 ^b^	0.053 ^b^	0.061	0.000
Sad	LD	0.022 ^a^	0.063 ^b^	0.043 ^ab^	0.016	0.011
	HL	0.039 ^a^	0.014 ^b^	0.013 ^b^	0.011	0.017
Angry	LD	0.041 ^a^	0.015 ^b^	0.024 ^b^	0.009	0.004
	HL	0.027	0.014	0.015	0.009	n.s.
Surprised	LD	0.016	0.010	0.013	0.004	n.s.
	HL	0.008	0.009	0.009	0.003	n.s.
Scared	LD	0.002	0.003	0.003	0.001	n.s.
	HL	0.003	0.007	0.006	0.003	n.s.
Disgusted	LD	0.014	0.017	0.017	0.006	n.s.
	HL	0.023 ^a^	0.011 ^ab^	0.005 ^b^	0.007	0.007
Contempt	LD	0.007 ^a^	0.019 ^b^	0.013 ^ab^	0.005	0.009
	HL	0.023 ^a^	0.011 ^ab^	0.005 ^b^	0.002	0.007
Valence	LD	0.079 ^a^	0.014 ^ab^	−0.042 ^b^	0.042	0.005
	HL	−0.034 ^a^	0.012 ^ab^	0.021 ^b^	0.024	0.024
		Response to the odor Scale 0–1				
Neutral	LD	0.024 ^a^	0.870 ^b^	0.817 ^c^	0.019	0.000
	HL	0.811 ^a^	0.661 ^b^	0.767 ^a^	0.047	0.002
Happy	LD	0.839 ^a^	0.093 ^b^	0.183 ^b^	0.062	0.000
	HL	0.171 ^a^	0.149 ^ab^	0.061 ^b^	0.049	0.027
Sad	LD	0.036	0.041	0.052	0.012	n.s.
	HL	0.025 ^a^	0.043 ^ab^	0.049 ^b^	0.011	0.038
Angry	LD	0.024 ^a^	0.011 ^b^	0.019 ^ab^	0.007	0.043
	HL	0.016 ^a^	0.009 ^ab^	0.004 ^b^	0.004	0.007
Surprised	LD	0.014 ^a^	0.011 ^ab^	0.009 ^b^	0.002	0.012
	HL	0.009	0.006	0.008	0.003	n.s.
Scared	LD	0.005 ^a^	0.002 ^b^	0.004 ^ab^	0.002	0.023
	HL	0.003	0.005	0.005	0.002	n.s.
Disgusted	LD	0.006	0.049	0.018	0.025	n.s.
	HL	0.015 ^a^	0.001 ^b^	0.001 ^b^	0.004	0.003
Contempt	LD	0.020	0.028	0.028	0.006	n.s.
	HL	0.009 ^a^	0.004 ^b^	0.002 ^b^	0.001	0.000
Valence	LD	0.125 ^a^	0.106 ^a^	−0.059 ^b^	0.033	0.000
	HL	−0.011 ^a^	0.090 ^b^	0.005 ^a^	0.041	0.016
		Response to the taste Scale 0–1				
Neutral	LD	0.806	0.843	0.821	0.028	n.s.
	HL	0.842	0.810	0.793	0.028	n.s.
Happy	LD	0.032	0.015	0.016	0.014	n.s.
	HL	0.006 ^a^	0.033 ^ab^	0.040 ^b^	0.017	0.046
Sad	LD	0.023	0.018	0.024	0.007	n.s.
	HL	0.016	0.028	0.024	0.009	n.s.
Angry	LD	0.046	0.036	0.037	0.010	n.s.
	HL	0.041	0.039	0.034	0.012	n.s.
Surprised	LD	0.018	0.013	0.018	0.005	n.s.
	HL	0.012	0.015	0.019	0.005	n.s.
Scared	LD	0.002	0.003	0.004	0.001	n.s.
	HL	0.004	0.003	0.002	0.001	n.s.
Disgusted	LD	0.021 ^ab^	0.015 ^a^	0.025 ^b^	0.050	0.045
	HL	0.023	0.025	0.024	0.007	n.s.
Contempt	LD	0.007	0.004	0.005	0.002	n.s.
	HL	0.004	0.009	0.010	0.005	n.s.
Valence	LD	−0.042	−0.045	−0.057	0.020	n.s.
	HL	−0.064	−0.049	−0.046	0.018	n.s.

Note: ^1^ LD, *Longissimus dorsi*; HL, hind leg. ^2^ SCD, standard compound diet; CG4, standard compound diet + 4% *C. glomerata* biomass; CG8, standard compound diet + 8% *C. glomerata* biomass. ^3^ The means with distinct superscript letters (a–c) in a row differ significantly (*p* < 0.05). ^4^ Means with ab superscript letters in a row did not have significant differences between groups (*p* > 0.05). ^5^ 0 means that no emotion is expressed at all, and 1 means the maximum value of the fitted model (for valence, a scale of −1 to 1). ^6^ SEM, standard error of the means. ^7^ n.s., not significant (*p* > 0.05).

## Data Availability

Not applicable.

## Supplementary Materials

The following supporting information can be downloaded at: https://www.mdpi.com/article/10.3390/ani13132179/s1, Table S1: Ingredients in rabbit feed and chemical composition of a standard compound diet and diets supplemented with different dosages of *C. glomerata* biomass (52–122 days old).

## References

[1] OECD (2015). Food and Agriculture Organization of the United Nations OECD-FAO Agricultural Outlook 2015.

[2] Altmann B.A., Neumann C., Velten S., Liebert F., Mörlein D. (2018). Meat Quality Derived from High Inclusion of a Micro-Alga or Insect Meal as an Alternative Protein Source in Poultry Diets: A Pilot Study. Foods.

[3] Shaaban M.M., Kholif A.E., Abd El Tawab A.M., Radwan M.A., Hadhoud F.I., Khattab M.S.A., Saleh H.M., Anele U.Y. (2021). Thyme and celery as potential alternatives to ionophores use in livestock production: Their effects on feed utilization, growth performance and meat quality of Barki lambs. Small Rumin. Res..

[4] Ghasemi-Sadabadi M., Ebrahimnezhad Y., Maheri-Sis N., Shaddel Teli A., Ghiasi Ghalehkandi J., Veldkamp T. (2021). Supplementation of pomegranate processing waste and waste soybean cooking oil as an alternative feed resource with vitamin E in broiler nutrition: Effects on productive performance, meat quality and meat fatty acid composition. Arch. Anim. Nutr..

[5] Altmann B.A., Wigger R., Ciulu M., Mörlein D. (2020). The effect of insect or microalga alternative protein feeds on broiler meat quality. J. Sci. Food Agric..

[6] Pethick D.W., Ball A.J., Banks R.G., Hocquette J.F. (2011). Current and future issues facing red meat quality in a competitive market and how to manage continuous improvement. Anim. Prod. Sci..

[7] Petracci M., Soglia F., Leroy F. (2018). Rabbit meat in need of a hat-trick: From tradition to innovation (and back). Meat Sci..

[8] Nutautaitė M., Vilienė V., Racevičiūtė-Stupelienė A., Bliznikas S., Karosienė J., Koreivienė J. (2022). Cladophora glomerata as a potential nutrient source in animal nutrition. Proceedings of the 1st International PhD Student’s Conference at the University of Life Sciences in Lublin.

[9] Nutautaitė M., Racevičiūtė-Stupelienė A., Bliznikas S., Jonuškienė I., Karosienė J., Koreivienė J., Vilienė V. (2022). Evaluation of Phenolic Compounds and Pigments in Freshwater Cladophora glomerata Biomass from Various Lithuanian Rivers as a Potential Future Raw Material for Biotechnology. Water.

[10] Nutautaitė M., Vilienė V., Racevičiūtė-Stupelienė A., Bliznikas S., Karosienė J., Koreivienė J. (2021). Freshwater Cladophora glomerata Biomass as Promising Protein and Other Essential Nutrients Source for High Quality and More Sustainable Feed Production. Agriculture.

[11] Nutautaitė M., Racevičiūtė-Stupelienė A., Bliznikas S., Vilienė V. (2023). Enhancement of Rabbit Meat Functionality by Replacing Traditional Feed Raw Materials with Alternative and More Sustainable Freshwater Cladophora glomerata Macroalgal Biomass in Their Diets. Foods.

[12] Sasaki K. (2022). Diversity of Japanese consumers’ requirements, sensory perceptions, and eating preferences for meat. Anim. Sci. J..

[13] Pethick D.W., Hocquette J., Scollan N.D., Dunshea F.R. (2021). Review: Improving the nutritional, sensory and market value of meat products from sheep and cattle. Animal.

[14] Arrington L.R., Cheeke P.R., Lebas F., Lebas S. (1977). Nutrient Requirements of Rabbits.

[15] Blasco A., Ouhayoun J. (2010). Harmonization of criteria and terminology in rabbit meat research. Revised proposal. World Rabbit. Sci..

[16] Honikel K.O. (2009). Water-holding capacity of meat. Muscle Development of Livestock Animals: Physiology, Genetics and Meat Quality.

[17] Zamorano J., Gambaruto M. (1997). Contribution to improving the meat water holding capacity test by the filter paper press method. A comparison of three methods for measuring areas. Meat Sci..

[18] Honikel K.O. (1998). Reference methods for the assessment of physical characteristics of meat. Meat Sci..

[19] Martínez-Álvaro M., Hernández P. (2018). Evaluation of the sensory attributes along rabbit loin by a trained panel. World Rabbit. Sci..

[20] Siddiqui S.A., Gerini F., Ikram A., Saeed F., Feng X., Chen Y. (2023). Rabbit Meat—Production, Consumption and Consumers’ Attitudes and Behavior. Sustainability.

[21] Wang J., Fan L., Zhou Q., Li J., Zhao P., Wang Z., Zhang H., Yan S., Huang L. (2018). Rapid Determination of Meat Moisture Content Using Radio-Frequency Dielectric Measurement. Access.

[22] Hafsa S.H.A., Khalel M.S., El-Gindy Y.M., Hassan A.A. (2021). Nutritional potential of marine and freshwater algae as dietary supplements for growing rabbits. Ital. J. Anim. Sci..

[23] Nollet L.M.L., Toldra F. (2009). Handbook of Processed Meats and Poultry Analysis.

[24] Varnam A.H., Sutherland J.P. (1995). The water of meat. Am. Lab..

[25] Warner R.D. (2017). Chapter 14-The Eating Quality of Meat—IV Water-Holding Capacity and Juiciness. Lawrie’s Meat Science.

[26] Hughes J.M., Oiseth S.K., Purslow P.P., Warner R.D. (2014). A structural approach to understanding the interactions between colour, water-holding capacity and tenderness. Meat Sci..

[27] Liu J., Arner A., Puolanne E., Ertbjerg P. (2016). On the water-holding of myofibrils: Effect of sarcoplasmic protein denaturation. Meat Sci..

[28] Kondjoyan A., Oillic S., Portanguen S., Gros J. (2013). Combined heat transfer and kinetic models to predict cooking loss during heat treatment of beef meat. Meat Sci..

[29] Czarniecka-Skubina E., Przybylski W., Jaworska D., Kajak-Siemaszko K., Wachowicz I. (2010). Effect of pH24 and intramuscular fat content on technological and sensory quality of pork. Pol. J. Food Nutr. Sci..

[30] Debrecéni O., Lípová P., Bučko O., Cebulska A., Kapelánski W. (2018). Effect of pig genotypes from Slovak and Polish breeds on meat quality. Arch. Für Tierz..

[31] Bocian M., Jankowiak H., Reszka P., Banaszak S. (2018). Pork quality with special emphasis on colour and its changes during storage. J. Cent. Eur. Agric..

[32] Strzyżewski T., Bilska A., Krysztofiak K. (2008). Correlation between pH value of meat and its colour. Sci. Nat. Technol..

[33] Karamucki T., Gardzielewska J., Jakubowska M., Rybak K., Garczewska J. (2013). The relationship between colour and pH in cold-stored quail breast muscle/Zależność między barwą a pH w mięśniach piersiowych przepiórek przechowywanych w warunkach chłodniczych. Ann. Anim. Sci..

[34] Jankowiak H., Cebulska A., Bocian M. (2021). The relationship between acidification (pH) and meat quality traits of polish white breed pigs. Eur. Food Res. Technol..

[35] Ribeiro D.M., Martins C.F., Costa M., Coelho D., Pestana J., Alfaia C., Lordelo M., de Almeida A.M., Freire J.P.B., Prates J.A.M. (2021). Quality Traits and Nutritional Value of Pork and Poultry Meat from Animals Fed with Seaweeds. Foods.

[36] Jerez-Timaure N., Sánchez-Hidalgo M., Pulido R., Mendoza J. (2021). Effect of Dietary Brown Seaweed (Macrocystis pyrifera) Additive on Meat Quality and Nutrient Composition of Fattening Pigs. Foods.

[37] King N.J., Whyte R. (2006). Does it look cooked?. A review of factors that influence cooked meat color. J. Food Sci..

[38] VAN Laack R.L.J.M., Berry B.W., Solomon M.B. (1996). Effect of Precooking Conditions on Color of Cooked Beef Patties. J. Food Prot..

[39] Liu D., Fan W., Xu Y., Yu S., Liu W., Guo Z., Huang W., Zhou Z., Hou S. (2021). Genome-wide association studies demonstrate that TASP1 contributes to increased muscle fiber diameter. Heredity.

[40] Schiaffino S., Dyar K.A., Ciciliot S., Blaauw B., Sandri M. (2013). Mechanisms regulating skeletal muscle growth and atrophy. FEBS J..

[41] Sarsenbek A., Wang T., Zhao J.K., Jiang W. (2013). Comparison of carcass yields and meat quality between Baicheng-You chickens and Arbor Acres broilers. Poult. Sci..

[42] Al-Soufi S., García J., Muíños A., López-Alonso M. (2022). Marine Macroalgae in Rabbit Nutrition—A Valuable Feed in Sustainable Farming. Animals.

[43] Aaslyng M.D., Jensen H., Karlsson A.H. (2018). The gender background of texture attributes of pork loin. Meat Sci..

[44] Fabre R., Dalzotto G., Perlo F., Bonato P., Teira G., Tisocco O. (2018). Cooking method effect on Warner-Bratzler shear force of different beef muscles. Meat Sci..

[45] Li S., Ma R., Pan J., Lin X., Dong X., Yu C. (2019). Combined effects of aging and low temperature, long time heating on pork toughness. Meat Sci..

[46] Desmet P.M.A., Schifferstein H.N.J. (2008). Sources of positive and negative emotions in food experience. Appetite.

[47] Hartwell H.J., Edwards J.S.A., Brown L. (2013). The relationship between emotions and food consumption (macronutrient) in a foodservice college setting-a preliminary study. Int. J. Food Sci. Nutr..

[48] Torrico D.D., Hutchings S.C., Ha M., Bittner E.P., Fuentes S., Warner R.D., Dunshea F.R. (2018). Novel techniques to understand consumer responses towards food products: A review with a focus on meat. Meat Sci..

