# Supplementary material for: Sensory Evaluation of Rabbit Meat from Individuals Fed Functional and More Sustainable Diets Enriched with Freshwater Cladophora glomerata Macroalgal Biomass
Source: Animals (Basel). 2023 Jul 3;13(13):2179. doi: 10.3390/ani13132179 (PMC10339916; doi:10.3390/ani13132179)
Supplement: Supplementary file 1 [file animals-13-02179-s001.zip › animals-2453996-supplementary.pdf]

Table S1. Ingredients in rabbit feed and chemical composition of a standard compound diet and diets supplemented with different dosages of C. glomerata biomass (52–122 days old).

	**Diet [3]**		
**Ingredient (%) [1,2]**	**SCD**	**CG4**	**CG8**
Corn	3.00	3.13	3.44
Barley	18.00	18.00	18.00
Oats	25.00	25.00	25.00
Sunflower meal	13.22	11.97	9.97
Linseed meal	1.00	1.00	1.00
Soy meal	3.72	3.00	3.00
Vegetable oil	1.00	1.00	1.00
Beer east	2.00	2.00	2.00
Hay	29.26	27.10	24.79
C. glomerata	–	4.00	8.00
Antimycotoxin	0.30	0.30	0.30
Vitamin-mineral premix	3.50	3.50	3.50
Total	100	100	100
	Chemical composition (%)		
DE (MJ/kg)	10.49	11.13	12.16
Crude protein	17.54	17.53	17.54
Crude fibre	13.56	14.39	15.05
Ash	10.03	10.37	10.31
Ether extract	3.12	3.20	3.10
NDF	32.49	34.19	35.89
ADF	19.71	20.12	20.73
ADL	4.89	4.94	5.15

Note: [1] Vitamin and mineral premix (per kg of feed): vitamin A 10.08 TV, vitamin D_3 1.14 TV, vitamin E 50.30 mg, vitamin K_3 0.99 mg, vitamin B_1 3.71 mg, vitamin B_2 2.80 mg, vitamin B_5 9.80 mg, vitamin B_{12} 0.01 mg, nicotinic acid 20.40 mg, folic acid 0.22 mg, choline chloride 170.00 mg, Mg 76.28 mg, Fe 317.00 mg, Zn 110.89 mg, Cu 19.16 mg, Co 0.29 mg, I 0.67 mg, Se 0.31 mg. [2] DE, diet energy; NDF, neutral detergent fibre; ADF, acid detergent fibre; ADL, acid detergent lignin. [3] SCD, standard compound diet; CG4, standard compound diet + 4% C. glomerate biomass; CG8, standard compound diet + 8% C. glomerata biomass.
